# Induced poverty, increased remittances: unveiling the lived realities of Nepali migrant workers

**DOI:** 10.1186/s12889-025-25503-0

**Published:** 2025-11-27

**Authors:** Animesh Ghimire, Mamata Sharma Neupane

**Affiliations:** 1 Sustainable Prosperity Initiative Nepal, Baneshwor-31, Kathmandu, Nepal; 2 School of Nursing and School of Public Health, Chitwan Medical College, Bharatpur-5, Kailashnagar, Chitwan Nepal

**Keywords:** Induced poverty, Migrant health, Labor exploitation, Remittances, Nepal

## Abstract

**Background:**

International labor migration from Nepal, primarily to Malaysia and Gulf Cooperation Council (GCC) states, is a significant economic driver, with remittances forming a substantial portion of the nation’s GDP. However, this economic reliance often overshadows the profound health consequences and conditions of “induced poverty”— encompassing debt, precarious living, and compromised well-being—experienced by migrant workers. Existing research predominantly focuses on pre-departure or returnee migrants, leaving a critical gap in understanding the health perspectives and lived realities of those currently employed in destination countries. This study aimed to explore how male Nepali migrant workers in Malaysia and GCC countries perceive and experience the impact of induced poverty and their labor conditions on their physical and mental health.

**Methods:**

Grounded in an interpretive paradigm, this qualitative descriptive study involved in-depth, semi-structured interviews with 17 male Nepali migrant workers currently employed in Malaysia and GCC countries. Initial participant recruitment utilized purposive and convenience sampling through self-registration via advertised flyers on a social media page, which was supplemented by snowball sampling to reach more participants. An overarching maximum variation strategy guided the final sample composition to ensure diverse perspectives. Data were analyzed inductively using applied thematic analysis.

**Results:**

Four interconnected themes emerged: (1) *The Body as a Site of Extraction*, detailing severe occupational hazards, physical deterioration, and the normalization of ill-health driven by economic imperatives; (2) *The Invisible Chains*, highlighting mental health erosion due to social isolation; (3) *The Remittance Trap*, revealing how pre-migration debt and ongoing financial precarity create a cycle of overwork and declining health despite remittance outflows; and (4) *Navigating Healthcare Deserts*, which exposed systemic barriers, perceived discrimination, and inadequate access to essential health services, forcing workers into detrimental coping strategies. Collectively, these themes reveal a stark reality where induced poverty systematically dismantles migrants’ health in real-time within destination countries, challenging prevailing narratives of migration as purely an economic uplift.

**Conclusion:**

This research concludes that conditions of induced poverty and exploitative labor practices endemic to the current migration system systematically compromise the health of Nepali migrant workers. Their firsthand accounts reveal an urgent need for a paradigm shift in migration governance, moving beyond a purely economic focus to prioritize the fundamental human rights, health equity, and dignity of migrant workers. This requires robust policy interventions in both Nepal and destination countries, focusing on ethical recruitment, stringent enforcement of labor protections, and accessible healthcare, to ensure migration becomes a pathway to genuine well-being rather than a precursor to suffering.

**Supplementary Information:**

The online version contains supplementary material available at 10.1186/s12889-025-25503-0.

## Background

International labor migration has become an indelible feature of the contemporary global economy, with millions of workers crossing borders, primarily from the Global South to the Global North [1], as well as to burgeoning economies within the South itself [2, 3]. This phenomenon is often framed by international development agencies and some academic discourse through the lens of a “triple-win” scenario [4], where sending countries benefit from remittances, receiving countries gain necessary labor, and migrants themselves achieve economic betterment. Remittances, in particular, have surged to become a dominant financial inflow for many low- and middle-income countries (LMICs), often surpassing official development assistance and foreign direct investment [5, 6]. However, this dominant economic narrative, while highlighting macroeconomic benefits, often inadequately accounts for the human and health costs borne by migrant workers, particularly those in low-skilled, precarious employment, challenging the very notion of a universally beneficial exchange [7, 8].

Nepal exemplifies a nation deeply integrated into this global labor migration system, having transformed into a significant labor-sending country over recent decades. It is estimated that well over 3.5 million Nepalis are working abroad [9], with the primary destinations being Malaysia, which has over 300,000 workers [10], and the Gulf Cooperation Council (GCC) states, which are estimated to host about 1.3 million workers [11]. Additionally, there is a significant migration to India, though accurate records are lacking due to the open border policy [12, 13]. The GCC comprises six member states: Bahrain, Kuwait, Oman, Qatar, Saudi Arabia, and the United Arab Emirates (UAE) [14]. The economic reliance on this labor export is undeniable, with remittances constituting over 26.6% of Nepal’s Gross Domestic Product, which amounts to approximately US$11 billion [15]. Yet, behind these significant economic figures lies a stark reality of systemic exploitation, hazardous working conditions, and severe health repercussions for a multitude of Nepali migrant workers [16, 17]. Furthermore, cross-border labor markets are strongly gendered: men are predominantly employed in construction and manufacturing, whereas women are concentrated in domestic and care work [3, 18]. Recognizing this gendered segmentation is essential to interpreting sector-specific exposures and the patterned health vulnerabilities that follow.

Crucially, female migrant workers constitute 18.72% of Nepal’s officially recorded migrant workforce [19]—a figure that does not capture the full extent of migration to India due to the open border [20]—and are disproportionately affected by exploitative practices. Their vulnerability often stems from their concentration in isolated sectors like domestic work, limited access to support networks, and entrenched gender norms that perpetuate power imbalances [21, 22]. Migrant Domestic Workers (MDWs) face specific and severe risks, including extreme isolation within private households, which heightens their susceptibility to wage theft, excessive working hours, psychological, physical, and sexual abuse, and human trafficking, often with little to no access to legal recourse or protection mechanisms within the destination country [21–24]. The kafala system, prevalent in many GCC states, further institutionalizes this vulnerability by tying a worker’s legal status directly to their employer, severely restricting their ability to change jobs or escape an abusive environment (Uçar, 2022). This creates what author Parreñas [25] terms a state of “unfreedom,” where workers are subject to the arbitrary power of their employers. This lived reality is compounded by the fact that women face a twofold challenge: navigating these exploitative labor conditions abroad and subsequently confronting significant social stigma upon return, particularly if they are unmarried or perceived by their communities to have engaged in work deemed “inappropriate” [26]. Study by Shrestha et al. [26] articulates this stigma through a participant’s account: “*In [Nepalese] society*,* they say that she earned money by going abroad and by having a bad relationship with a foreigner*.” This societal condemnation, often rooted in patriarchal norms, adds another layer of hardship to reintegration, a stark contrast to the “migrant hero” narrative often applied to male workers [27, 28].

These journeys undertaken by Nepali migrants, often initiated by acute domestic poverty and limited livelihood opportunities [29, 30], frequently leads to what this study terms “*induced poverty*” in the destination countries. “Induced poverty,” in this context, refers to a state of deprivation and vulnerability that is not a pre-existing condition alone but is actively created or exacerbated by the very processes and structures of labor migration. This includes crippling recruitment debts, wage exploitation, the high cost of living relative to earnings in host countries, and the financial burdens of unexpected health crises or family emergencies, all of which can trap migrants in cycles of precarity despite their employment [31–33]. This induced poverty manifests not only as financial hardship but also as a profound lack of health and well-being, as workers are frequently exposed to hazardous occupational environments, inadequate living conditions, and significant psychosocial stressors, often with limited access to sufficient healthcare or protective measures [34, 35]. This ongoing deterioration of health represents a critical, yet often under-acknowledged, dimension of the true cost of migration, challenging the narrative that remittances automatically translate into improved overall welfare for migrants and their families [36].

Despite a growing body of literature on Nepali labor migration, a significant gap persists in understanding the health perspectives and lived realities of Nepali migrants while they are currently residing and working in destination countries. Much of the existing research has focused on pre-departure vulnerabilities [9], the socio-economic impacts of remittances on families left behind [37, 38], or the health challenges faced by returnee migrants [39]. While valuable, these studies often do not capture the immediate, ongoing health experiences, coping mechanisms, and perceptions of risk and well-being from the standpoint of those actively embedded within the often-exploitative labor systems abroad. Addressing this critical void, the present research centers the voices and experiences of Nepali migrant workers currently working in Malaysia and the GCC states. Specifically, it addresses the in-situ health experiences of male Nepali migrant workers within these destinations, reflecting the gendered composition of our participant sample.

This study, through an exploration of their narratives, aims to provide a nuanced understanding of how the complex interplay of economic pressures, working conditions, social environment, and healthcare access in destination countries shapes their health and well-being. The central research question guiding this inquiry is: How do Nepali migrant workers employed in Malaysia and the GCCs perceive and experience the effects of “induced poverty” on their physical and mental health? Additionally, what strategies do they employ to navigate health challenges in these contexts?

## Methods

### Study design

This study was grounded in an interpretive paradigm, which seeks to understand the subjective meanings and lived experiences individuals construct within their specific socio-cultural contexts [40]. To achieve this, we employed an interpretive descriptive design, a methodology that moves beyond mere description to actively explore the meanings and explanations underlying participants’ experiences, with the explicit goal of generating practical, application-oriented insights [41]. This approach was deemed most suitable as our aim was not only to describe the health challenges faced by Nepali migrant workers but to interpret their experiences within the socio-economic context of “induced poverty,” thereby yielding findings with clear implications for policy and practice.

### Setting and participant recruitment strategy

This study focused on Nepali migrant workers currently employed in Malaysia and the Gulf Cooperation Council (GCC) countries. The initial stage of participant recruitment utilized a purposive sampling strategy [42] by targeting a large, private Facebook group known as a key online forum for this demographic. After the principal investigator (PI) contacted the group administrator, outlined the study’s objectives, and provided verification of ethical approval, the administrator facilitated recruitment by posting a study flyer in both Nepali and English. This flyer invited interested individuals who met the pre-defined inclusion criteria to self-register for the study, thus incorporating an element of convenience sampling [43] into this first phase of recruitment. Recruitment proved challenging in this context, which ultimately shaped the sample size and composition.

This flyer was designed to attract participants meeting specific inclusion criteria: (1) being Nepali citizens currently employed in Malaysia or a GCC country for a minimum of one year; (2) being 21 years of age or older; and (3) expressing willingness to share their experiences regarding work, health, and finances. The flyer detailed the study’s aims, provided information about the PI (AG), and included a QR code linking to an initial online screening questionnaire. This method allowed for a convenience sampling element, as individuals who self-identified as meeting the criteria and were interested could easily access the screening tool. Those who passed the initial screening were then provided with a plain language statement form, also in Nepali and English, detailing their rights and what participation would entail. Prospective participants were invited to contact the PI via email or WhatsApp [44] to express their interest; all who proceeded chose WhatsApp for initial contact. Gender was not an inclusion criterion; however, despite gender-neutral recruitment materials and outreach, subsequent recruitment outcomes resulted in a male-only sample.

This first phase of recruitment, conducted between January 2025 and April 2025, yielded 10 participants who self-registered through the screening process and were subsequently contacted and confirmed as eligible by the PI. Following this initial recruitment, and to enhance the diversity of experiences within our sample—particularly to ensure representation from various GCC countries, gender and with different lengths of stay—a snowball sampling technique was then employed [45]. The initial 10 participants were asked to recommend colleagues or acquaintances who also met the study’s inclusion criteria and might be willing to share their perspectives. They were explicitly encouraged to recommend female colleagues; however, the initial 10 participants reported that the women they approached declined to participate. Nevertheless, this referral process facilitated the recruitment of an additional seven participants. Throughout this secondary phase, we applied principles of maximum variation sampling [46] by selectively following up on referrals that would diversify our sample in terms of specific GCC countries, locations, and years of experience in the destination country. The PI meticulously verified that all 17 participants ultimately included in the study met the full inclusion criteria as per the approved research protocol.

### Data collection

Data were collected between January 2025 and May 2025 through in-depth, semi-structured interviews. The principal investigator, fluent in Nepali and English, conducted all interviews via secure video conferencing platforms at a time mutually convenient for the participants. Each interview lasted approximately 60 min. Prior to each interview, participants were provided with a plain-language information sheet outlining the study’s objectives, the voluntary nature of participation, confidentiality and anonymity safeguards, withdrawal rights, and data management procedures. Written informed consent was obtained electronically before the interview commenced, and audio recording proceeded only with explicit permission. Participants were offered the choice to be interviewed in Nepali or English; all participants opted for Nepali. The semi-structured interview guide was developed collaboratively by both authors, who are academics with extensive experience in public health and migration scholarship. The guide was informed by the research aims and a review of relevant literature and was designed to explore themes of occupational health, mental well-being, economic pressures, experiences of poverty, access to healthcare, and coping strategies. The English language version of the semi-structured interview guide developed for this study is available as Supplementary File 1.

All audio recordings were transcribed verbatim in Nepali by the principal investigator and then translated into English. To ensure translational accuracy and conceptual equivalence, the second author (MSN), who is also fluent in Nepali and English, independently reviewed and cross-checked the translated transcripts against the original Nepali audio. All transcripts were de-identified using codes (P1 to P17) to protect participant anonymity.

### Data analysis

We employed an applied thematic analysis approach to analyze the transcribed interview data outlined by Guest et al. [47]. This iterative process allowed themes to emerge directly from the participants’ narratives, ensuring that their perspectives remained central to the analysis. The initial phase involved data familiarization, where both researchers independently read the transcripts multiple times, alongside contemporaneous field notes, to immerse themselves in the data. Subsequently, initial codes were systematically generated across the dataset by both authors, identifying salient features relevant to the research questions concerning induced poverty, health, and remittance pressures. These codes captured both semantic and latent meanings. Codes were then collated into sub‑categories and broader analytic categories; through regular analytic meetings, we reviewed and refined candidate themes against coded extracts and the full dataset, and defined and named the final themes. The Findings present the resulting analytic narrative, interweaving illustrative participant quotations with thematic interpretation and explicitly linking back to the research questions (Table 1).

**Table 1:** Example of data analysis process

Meaning Unit (Participant’s Quotes)	Code	Sub-categories	Categories	Theme
P12 (Qatar): “…safety is less important because we are from Nepal, we are cheaper to replace.”	Perceived disposability, Differential safety	Discriminatory safety practices, Devaluation of Nepali workers	Occupational Hazards & Discrimination	1. The Body as a Site of Extraction: Occupational Hazards, Health Erosion, and Suffering
P9 (Saudi Arabia): “…Being sick is a luxury none of us can afford here. Our bodies are just tools for the company…”	Sickness as luxury, Body as tool	Economic coercion over health, Dehumanization, Normalization of ill-health	Health Neglect due to Economic Pressure	1. The Body as a Site of Extraction: Occupational Hazards, Health Erosion, and Suffering
P1 (Malaysia): “…Weeks pass without speaking to anyone… At night, the silence is heavy… heart feels like it will burst with sadness.”	Profound loneliness, Lack of social contact	Social isolation, Emotional distress, Lack of support systems	Psychosocial Impact of Isolation	2. The Invisible Chains: Mental Health Erosion and Social Isolation
P13 (Qatar): “Every riyal I earn is already counted for – loan repayment, children’s school fees… The tension is always there… Sometimes I cannot sleep…”	Financial worry, Remittance pressure	Constant financial anxiety, Impact on sleep, Pressure of obligations	Economic Stress & Mental Burden	2. The Invisible Chains: Mental Health Erosion and Social Isolation
P4 (Malaysia): “…family took a loan… Every month, a significant part of my salary goes to pay that interest… I eat less… every ringgit I save must go to that loan first.”	Pre-migration debt, Sacrificing needs	Debt bondage, Impact of interest rates, Deprioritizing personal well-being	Debt-Driven Deprivation	3. The Remittance Trap: Debt, Economic Insecurity, and the Erosion of Health
P6 (Malaysia): “…had to send extra money for his hospital bills. I had to borrow again here… Now I have two loans to pay. I work more overtime, even when my body is screaming for rest.”	Compounding debt, Overwork for remittances	Cycle of borrowing, Health sacrifice for financial needs, Remittance demands	Protracted Precarity & Health Decline	3. The Remittance Trap: Debt, Economic Insecurity, and the Erosion of Health
P11 (Saudi Arabia): “…big hospital, but that is very expensive… So, I just prayed and took the local medicine friends gave me.”	Cost as barrier, Informal coping	Financial barriers to healthcare, Reliance on non-medical solutions	Inaccessible Healthcare & Coping Mechanisms	4. Navigating Healthcare Deserts: Systemic Barriers, Exclusion, and Strategies of Health Resilience
P2 (Malaysia): “…they see you are a Nepali worker, the way they look at you… it’s different… They think we are just laborers; our health is not important.”	Perceived discrimination, Devalued health	Discriminatory healthcare experiences, Feeling of being second-class	Exclusion & Discriminatory Treatment in Healthcare	4. Navigating Healthcare Deserts: Systemic Barriers, Exclusion, and Strategies of Health Resilience

### Rigor and trustworthiness

To ensure the trustworthiness of our findings, we adhered to established criteria for qualitative rigor, including credibility, dependability, confirmability, and transferability [48].

Credibility was enhanced through prolonged engagement during the data collection period and member checking. Summaries of key anonymized findings and illustrative quotes were shared with a subset of participants (those who agreed to be re‑contacted) for their feedback on the accuracy and resonance of our interpretations; their affirmations and minor clarifications were incorporated. Dependability was addressed through a transparently documented research process supported by a dated audit trail of sampling, coding, and theme‑development decisions, with regular analytic meetings recorded to capture major interpretive shifts.

Confirmability was promoted through investigator triangulation (both authors involved in analysis and interpretation) and reflexivity. We engaged in ongoing reflexive discussions, critically examining our positionalities as healthcare clinicians and academics embedded in Nepal’s healthcare system and migration scholarship, and how these might influence interpretation. Finally, transferability is supported by a rich description of the study context, recruitment methods, data collection, and analytic approach, with participant characteristics reported in Table 2 to aid readers in assessing applicability to similar populations.

### Ethical considerations

Ethical approval for this study was obtained from the Institutional Review Board (IRB) of Chitwan Medical College (Approval Number: CMC-IRC/080/081–111) and the Nepal Health Research Council (NHRC) (Approval Number: 348/2024), prior to initiating participant recruitment. All participants were provided with a detailed plain language statement (in Nepali and English) outlining the study's purpose, procedures, voluntary nature of participation, potential risks and benefits, confidentiality measures, and data usage. Written informed consent was secured from each participant before interviews. It was emphasized that they could withdraw at any time without penalty. To protect anonymity, all identifying information was removed from transcripts, and participant codes (P1-P17) are used in this manuscript.

## Results

### Participant characteristics

A total of 17 male Nepali migrants participated in this study. At the time of the interviews, all participants were engaged in a range of occupations listed in Table 2. Eight participants (P1-P8) were residing and working in Malaysia. The remaining nine participants were located in GCC countries: three in Saudi Arabia (P9-P11), two in Qatar (P12-P13), two in Kuwait (P14-P15), and two in the UAE (P16-P17). Regarding their origin within Nepal, 15 participants reported coming from rural backgrounds, while two were from urban areas. The highest level of education achieved by the participants was high school (Grade 12 equivalent), and the lowest was secondary school (Grade 7). The duration of their employment in their respective destination countries at the time of the interview was a minimum of one year, with the specific length of stay varying among participants. Further socio-demographic details of the participants are summarized in Table 2.

**Table 2:** Socio-demographic characteristics of participants (*N* = 17)

Participant ID	Sex	Age	Occupation (at time of interview)	Country of Destination	Origin in Nepal (Urban/Rural)	Highest Level of Education	Marital Status	Years in Destination Country
P1	Male	26	Agriculture	Malaysia	Urban	High School (Grade 12 pass)	Married	3
P2	Male	24	Construction/farming	Malaysia	Rural	High School (Grade 10 pass)	Unmarried	2
P3	Male	29	Hospitality	Malaysia	Rural	High School (Grade 12 pass)	Married	5
P4	Male	23	Cleaner/Security	Malaysia	Rural	Secondary School (Grade 8 pass)	Married	1
P5	Male	30	Manufacturing/factory-based	Malaysia	Rural	High School (Grade 12 pass)	Married	6
P6	Male	28	Driver	Malaysia	Rural	High School (Grade 10 pass)	Married	4
P7	Male	22	Construction/farming	Malaysia	Rural	High School (Grade 12 pass)	Married	2
P8	Male	31	Factory Work	Malaysia	Rural	High School (Grade 12 pass)	Married	7
P9	Male	27	Heavy Machine Operator	Saudi Arabia	Urban	High School (Grade 12 pass)	Married	4
P10	Male	21	Cleaner/Security	Saudi Arabia	Rural	High School (Grade 10 pass)	Unmarried	1
P11	Male	27	Driver	Saudi Arabia	Rural	High School (Grade 12 pass)	Married	5
P12	Male	24	Construction/farming	Qatar	Rural	High School (Grade 10 pass)	Unmarried	3
P13	Male	25	Hospitality	Qatar	Rural	High School (Grade 12 pass)	Married	2
P14	Male	26	Cleaner/Security	Kuwait	Rural	High School (Grade 10 pass)	Married	6
P15	Male	28	Manufacturing/factory-based	Kuwait	Rural	High School (Grade 12 pass)	Married	3
P16	Male	22	Construction/farming	UAE	Rural	Secondary School (Grade 7 pass)	Unmarried	1
P17	Male	34	Hospitality	UAE	Rural	High School (Grade 12 pass)	Married	5

### Findings

**Fig. 1:**
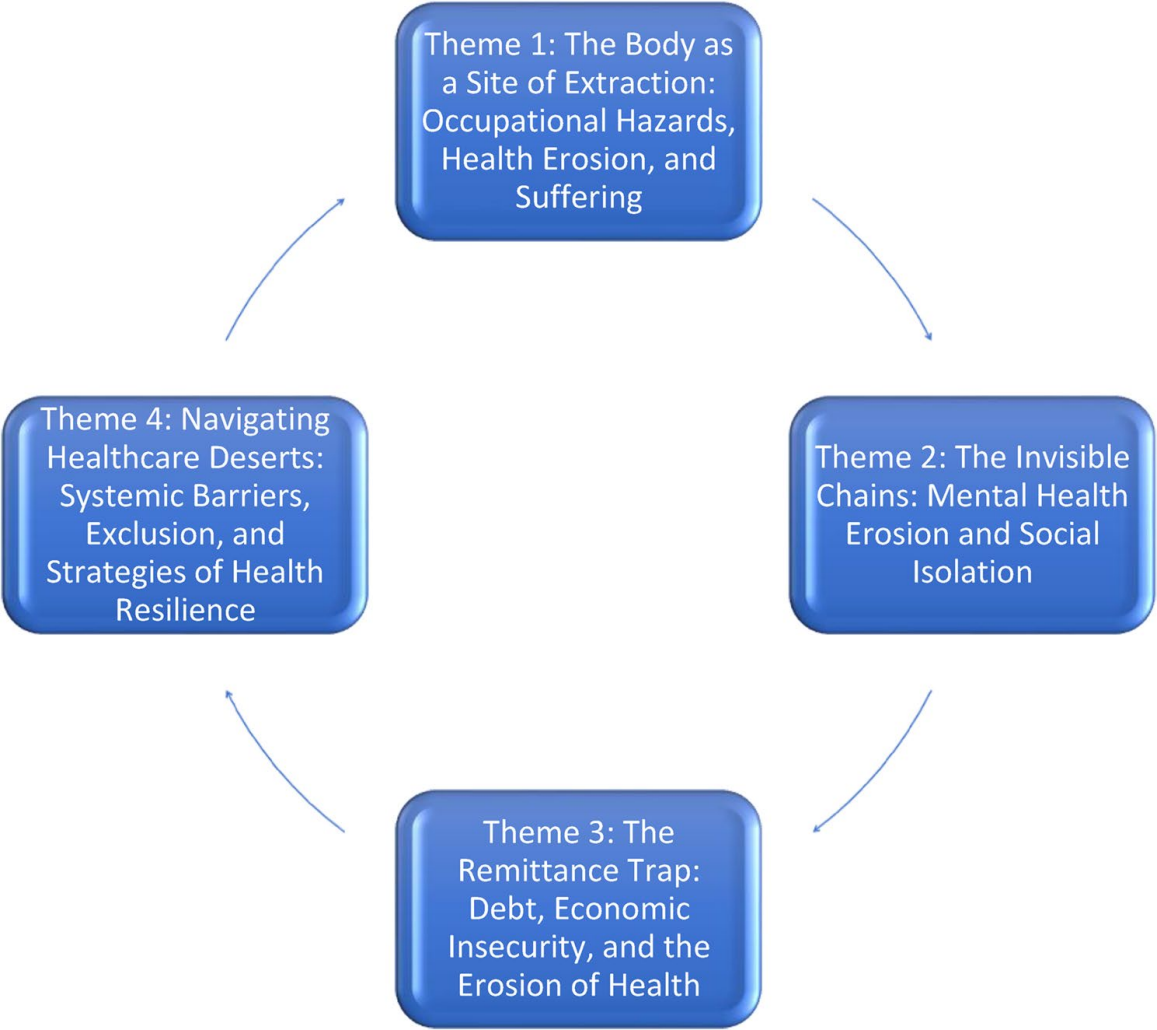
A thematic map illustrating the interconnected dimensions of induced poverty and its health impacts on Nepali migrant workers

Our analysis of the lived experiences of Nepali migrant workers in Malaysia and the GCC reveals a profound disconnect between the aspiration for economic betterment and the reality of their deteriorating well-being. Four interconnected themes emerged from their narratives, collectively illuminating the multifaceted nature of “induced poverty” and its detrimental health impacts (Fig. 1). These themes map a trajectory of suffering, beginning with the *physical extraction* from the body through hazardous labor, extending to the erosion of *mental health* under the weight of social isolation, deepening with the paradoxical economic precarity of the *remittance trap*, and culminating in the struggle to navigate *exclusionary healthcare systems* in destination countries.

#### Theme 1: the body as a site of extraction: occupational hazards, health erosion, and the normalization of suffering

Our research with participants uncovers a stark and consistent narrative wherein their bodies become primary sites of economic extraction, often at the expense of profound and lasting physical harm. Participants’ accounts vividly detail how the relentless pursuit of livelihoods abroad is inextricably linked to severe occupational hazards, a gradual yet debilitating erosion of physical health, and, alarmingly, a deeply ingrained normalization of illness and pain. These embodied costs are not mere unfortunate byproducts of migration but are revealed as systemic outcomes of labor conditions where worker well-being is systematically deprioritized in the face of economic imperatives.

The daily confrontation with perilous working conditions was a dominant sub-theme. While major construction sites in some host countries may appear to have modern infrastructure, participants reported that the safety equipment provided to them, as Nepali workers, often felt substandard or insufficient compared to that given to workers of other nationalities, or that safety protocols were selectively enforced. P12, a construction worker in Qatar, described this subtle but significant disparity:

*“It’s clear there’s a difference in how we’re treated regarding safety. We observe other workers*,* often from different nationalities*,* receiving better quality safety equipment like new helmets and strong boots. For us Nepali workers*,* the gear provided is sometimes old*,* or it seems more for appearance than actual protection – ‘just for show*,*’ as they say. While supervisors might talk about safety*,* when there’s pressure to complete work quickly*,* those safety concerns often seem to be overlooked*,* and risks are taken. This makes us feel that our safety is considered less important*,* perhaps because*,* as workers from Nepal*,* we are perceived as being more expendable or ‘cheaper to replace’ if an accident occurs.”*

P12’s account of differential treatment in safety provision reveals a nuanced form of discrimination that directly heightens physical risk. This lived experience of being devalued, rooted in his migrant status and country of origin, is a profound psychosocial stressor that compounds the tangible dangers of his work, illustrating a direct link between perceived discrimination and adverse health outcomes.

Beyond the immediate threat of accidents, a more insidious process of physical deterioration was described by many participants, stemming from the cumulative impact of years of arduous labor and exposure. P5, employed in a Malaysian factory for six years, detailed this slow decline:

*“My body is not the same. Before*,* I could work 14 hours*,* no problem. Now*,* after 7–8 hours*,* my back feels like it will break*,* and my joints ache all night. The dust in the factory*,* the fumes […] sometimes it’s hard to breathe properly even with the mask. We all know this work is eating us from the inside*,* but the debt back home […] doesn’t wait for your body to heal.”*

This narrative from P5 poignantly illustrates the insidious erosion of health capital. It highlights a critical finding from those currently embedded in these labor processes: the conscious, yet constrained, decision-making where long-term health is continuously sacrificed for immediate economic survival and remittance obligations.

The physical burden is further compounded by precarious living conditions and inadequate nutrition, which participants consistently linked to recurrent illnesses. P16, residing in a labor residential housing in the UAE, shared his experience:

*“Twelve of us live in a room meant for maybe six. The toilets are few and often dirty. The food the company provides is mostly rice and a watery lentil soup. How can a man build strength on that to work 12 hours in the sun? We fall sick often – fevers*,* diarrhea*,* skin rashes. You go to the company’s clinic; they give you paracetamol for everything. Real treatment costs money because we have to go to the big hospitals. However*,* I don’t say anything because I do not want to spend money […]*,* the company will take us to the bigger hospitals if need be*,* but they will also deduct the medical cost from our salary*,* and I need to save every paisa [cent].”*

P16’s account vividly demonstrates the intersection of low wages, exploitative living arrangements, and compromised health. This perspective from a worker currently enduring these conditions provides crucial evidence of how the very infrastructure of labor migration can systematically undermine health. Perhaps the most disturbing finding is the pervasive normalization of pain and the suppression of health needs, driven by economic coercion. P9, a heavy machinery operator in Saudi Arabia, offered a chilling insight:

*“Here*,* if you say you are sick*,* they look at you like you are trying to escape work. Small injuries*,* constant pain […]*,* you learn to live with it. If you go to the supervisor too many times*,* you might lose your job*,* and then how will your family eat [back home]? So*,* you swallow the pain*,* take some medicine you brought from Nepal*,* and pray it does not get worse. Being sick is a luxury none of us can afford here. Our bodies are just tools for the company; when one tool breaks*,* they will get another.”*

P9’s stark words reveal a profound instrumentalization of the migrant body within these labor regimes. His perception of being a replaceable ‘tool’ underscores how workers are compelled to suppress their health needs as a condition of employment. This normalization of suffering is not a passive acceptance but an active, albeit coerced, strategy for survival, directly driven by job insecurity and the overwhelming pressure to remit. The sentiment that ‘sickness is a luxury’ powerfully illustrates how their economic precarity dictates health priorities, forcing individuals to deprioritize their well-being to maintain their livelihoods.

#### Theme 2: the invisible chains: mental health erosion and social isolation

Beyond the visible physical injuries and deterioration, our research uncovers the often silent erosion of mental well-being among Nepali migrant workers. Participants described a pervasive sense of psychological distress stemming from social isolation, the immense pressure of familial and financial obligations, and the constant anxieties associated with their employment and living conditions. These “invisible chains” significantly impact their overall health, revealing how the economic imperative of migration can lead to severe mental health burdens, often unaddressed and unacknowledged both by employers and support systems.

The loneliness and isolation experienced in foreign lands, far from familial and community support systems, was a recurring sub-theme. P1, working in a remote agricultural plantation in Malaysia, shared his feelings of despair:

*“Here*,* it is just work and the small room. Weeks pass without speaking to anyone beyond a few words with the supervisor. At night*,* the silence is heavy. I think of my children*,* my wife… are they okay? Are they safe? Sometimes*,* the heart feels like it will burst with sadness. There is no one to share these feelings with.”*

P1’s account underscores the deep emotional cost of separation, a common experience for migrants but one whose intensity and impact are vividly captured by those currently living it. While the economic benefits of remittances are often highlighted, his words reveal the hidden emotional labor and the significant mental health strain that underpins these financial flows, a perspective that adds a critical layer to understanding the actual “cost” of migration from the standpoint of those enduring it.

The relentless pressure to earn and remit, often driven by pre-existing poverty and debts incurred for migration, emerged as a major source of anxiety and stress. P13, working in Qatar’s service industry, described this constant worry:

*“Every riyal [Qatari currency] I earn is already counted for – loan repayment*,* children’s school fees*,* parents’ medicine*,* household expenses. If I fall sick for a few days*,* or if the overtime is cut*,* the calculation goes wrong. The tension is always there. Sometimes I cannot sleep*,* just thinking about how to manage everything. The family back home only sees the money; they don’t see the sleepless nights here.”*

This testimony from P13 highlights the immense psychological burden tied directly to the economic expectations of migration. The “remittance pressure” he describes illustrates how financial obligations can transform into a significant mental health stressor, a direct link between the study’s focus on “induced poverty” and health outcomes. This ongoing, lived anxiety is a critical finding from current migrants, offering a more immediate understanding than retrospective accounts of financial stress.

For some, the combination of harsh working conditions, isolation, and financial worries culminates in more severe mental health challenges, including depression and suicidal ideation. P8, a factory worker in Malaysia who had witnessed a co-worker attempt suicide, spoke in hushed tones:

*“It’s not easy here. Some days*,* the mind just gives up. You feel trapped*,* like there is no way out. We see colleagues who stop talking*,* who just stare blankly. The man who tried to end his life […] he was quiet for weeks. He had a big loan*,* and his wife was sick. The company did nothing for him. We are just numbers to them. Sometimes*,* you feel like less than human.”*

P8’s account is a stark reminder of the extreme psychological distress that can result from the confluence of exploitative labor practices and social isolation. His observation about workers feeling “less than human” points to the dehumanizing aspects of their experience, which directly impacts mental health. This finding, from someone witnessing these severe outcomes firsthand, provides a crucial, often underreported, perspective on the gravest mental health consequences of migration, moving beyond general statistics on depression to the lived reality of despair within these communities.

The lack of accessible and culturally appropriate mental health support in destination countries was a consistent complaint, leaving workers to cope with their distress in isolation or through informal, often inadequate, means. P17, working in the UAE, explained:

*“If your mind is sick*,* where do you go? The company doctor is for the body*,* not the mind. And even if there was someone*,* how do you explain in a foreign language what is happening inside you? We talk among ourselves*,* try to give each other courage. But sometimes*,* the sadness is too big for a friend’s words.”*

This statement from P17 highlights a critical systemic failure: the near-total absence of mental health infrastructure tailored to the needs of migrant workers. This lack of support directly exacerbates their vulnerability, forcing them to internalize their struggles or rely solely on peer support, which, while valuable, is often insufficient to address serious mental health conditions.

#### Theme 3: the remittance trap: debt, economic Insecurity, and the erosion of health

While remittances are often lauded as a cornerstone of Nepal’s economy and a lifeline for migrant families, our research with Nepali workers reveals a more complex and often pernicious reality: a “remittance trap.” This trap is characterized by a cycle of debt incurred to facilitate migration, persistent economic insecurity despite regular earnings, and a consequent erosion of both physical and mental health. Participants’ narratives compellingly illustrate how the very act of migrating to send money home can, paradoxically, deepen financial precarity and induce new forms of poverty that directly compromise their well-being.

A significant number of participants reported being ensnared in substantial debt even before earning their first salary abroad, primarily due to exorbitant recruitment fees and travel costs. This initial indebtedness creates immense pressure to remit, often at the expense of their own basic needs and health. P4, who works as a cleaner in Malaysia, explained the weight of this burden:

*“Before I even came here*,* my family took a loan of nearly five lakh rupees (approx. USD 3600). The interest is high. Every month*,* a significant part of my salary pays that interest and the loan. I eat less*,* I don’t buy new clothes*,* I don’t even go to the doctor if I am sick*,* because every ringgit [Malaysian currency] I earn needs to go towards the loan.”*

P4’s experience highlights how pre-migration debt fundamentally shapes the migrant’s economic reality and health-seeking behaviors. This finding, from a worker currently grappling with such debt, offers a crucial perspective on how “induced poverty” begins even before migration, creating a cycle where health is immediately deprioritized.

The pressure to maximize remittances, often to service these debts or meet escalating family needs, frequently led to workers enduring exploitative wage practices, further entrenching their economic insecurity and impacting their health. P10, working in Saudi Arabia, described the constant struggle:

*“The company promised a certain salary*,* but after deductions for food*,* accommodation*,* and sometimes unexplained ‘fees*,*’ what we get in hand is much less. Overtime is often not paid correctly. But we cannot complain too much. If we lose this job*,* how will we pay the loans? How will our children go to school? So*,* we accept it. This stress*,* this worry about money*,* it never leaves. It affects my sleep*,* my appetite. I am always tired.”*

This testimony from P10 illustrates the direct link between wage exploitation, a consequence of their vulnerable position, and both economic precarity and mental health erosion. Furthermore, the expectation of continuous remittances, regardless of the worker’s own circumstances or health status in the destination country, creates a significant psychosocial burden. P15, working in Kuwait, shared his frustration:

*“Even when I was sick with a fever for a week and could not work*,* my family called asking for money for a festival. They don’t understand how hard it is here*,* or how little we sometimes have left after sending everything. It feels like I am just a money-making machine for them. Sometimes I feel so empty*,* like my own life*,* my own health*,* doesn’t matter[…] as long as the money keeps going home.”*

P15’s poignant statement reveals the emotional and psychological dimensions of the remittance trap. The pressure to fulfill familial expectations, often without a full understanding from those back home of the hardships endured, contributes to feelings of alienation and diminishes self-worth.

The cyclical nature of debt and the constant struggle to remit often forced participants into a state of protracted precarity, where any unexpected expense, such as a medical emergency or a family crisis back home, could plunge them deeper into financial distress, further compromising their health. P6, working in Malaysia, recounted such an experience:

*“My son fell ill back in Nepal*,* and I had to send extra money for his hospital bills. I had to borrow again here*,* from friends. Now I have two loans to pay. I work more overtime*,* even when my body is screaming for rest. Sometimes I feel like I will never escape this cycle. My health is getting worse*,* but the debts are getting bigger.”*

P6’s situation exemplifies how the remittance economy, rather than providing a straightforward escape from poverty, can create new layers of indebtedness and vulnerability for the migrant worker. This “remittance trap,” where the need to remit perpetuates a cycle of overwork, debt, and declining health, presents a critical finding that questions the broader concept that the migration of Nepali workers has made the country and the migrants’ families prosperous [49]—but at what cost?

#### Theme 4: navigating healthcare deserts: systemic barriers, exclusion, and strategies of health resilience

Beyond the direct occupational hazards and the normalization of ill-health, our findings illuminate the challenges Nepali migrant workers face in accessing adequate healthcare in destination countries. Participants described navigating what can best be termed “healthcare deserts”—environments characterized by significant systemic barriers, experiences of exclusion, and a glaring lack of culturally and financially accessible health services. In response, workers often develop informal strategies of resilience further highlighting the health impacts of their induced poverty and precarious legal status.

A primary barrier to healthcare access was the prohibitive cost of medical services. P11, working in a construction company in Saudi Arabia, shared his dilemma when he fell seriously ill:

*“I had a high fever*,* severe stomach and body pain for many days. The company clinic only gave basic medicine. They said if it gets serious*,* I have to go to the big hospital*,* but that will be expensive […]. I ended up not going to the hospital and relied on the medicines given by company and my friends. It was a frightening time*,* but I saved money and didn’t waste it.”*

P11’s experience, common among participants, demonstrates how financial precarity directly translates into deferred or foregone medical care. This finding, from a worker currently facing such a choice, starkly illustrates how induced poverty acts as an insidious driver of healthcare inequity, forcing individuals to gamble with their health due to economic necessity. The “choice” to avoid expensive treatment, even when seriously ill, is a direct consequence of their low-income status and the overriding obligation to support their families, a critical aspect of the health-poverty nexus in migration.

Beyond cost, participants reported experiences of discriminatory treatment or a perception of receiving substandard care due to their nationality and socio-economic status. P2, working in Malaysia, recounted his experience at a clinic:

*“When you go to some clinics*,* and they see you are a Nepali worker*,* the way they look at you*,* the way they talk […] it’s different. Sometimes you wait for hours*,* and then the doctor sees you for only two minutes. You feel like they don’t really care about your problem. They think we are just laborers; our health is not important. It makes you not want to go even if you are sick.”*

This perception of being treated as “second-class” within healthcare institutions, as articulated by P2, acts as a significant deterrent to seeking care. The lack of linguistically and culturally appropriate health services further compounded these challenges. Many participants struggled to communicate their symptoms effectively or understand medical advice provided in a foreign language. P17, from the UAE, explained:

*“The doctors here speak Arabic or English*,* and they speak very fast. It is hard to explain exactly how I am feeling or what the problem is. Sometimes*,* they give medicine*,* but I don’t fully understand how to take it or what it is for. There are no translators for Nepali. You just nod your head and hope for the best. It’s very difficult when you are sick […]”*.

P17’s account underscores the critical role of language and cultural competency in healthcare delivery, a factor often overlooked in the provision of services to migrant populations. In the face of these formidable barriers, workers often resorted to self-medication, reliance on informal advice from their colleagues, or delaying treatment until their return to Nepal, often with severe consequences. P6, who developed a chronic cough while working in Malaysia, shared his regret:

*“I had this cough for months*,* but I kept taking cough syrup from the small shops. Going to a proper doctor was too much money and too much hassle with the company due to paperwork […]. I thought it would go away. When I finally went back to Nepal*,* the doctor said it was a serious lung infection and I should have come much earlier. The treatment was long and expensive*,* and I could not return to Malaysia for more than a year.”*

P6’s experience reveals that what might appear as health ‘resilience’ is, in reality, a form of deferred suffering compelled by systemic constraints. This reliance on inadequate coping mechanisms, a choice born of necessity, often leads to the exacerbation of health problems, transforming treatable conditions into chronic illnesses. Capturing this decision-making process as it occurs in the destination country provides a critical perspective. It demonstrates how systemic barriers to timely healthcare directly contribute to long-term negative health outcomes that entrench a cycle of poverty and ill-health, a burden that follows the migrant upon their return and is central to understanding the full trajectory of the migration-health nexus.

## Discussion

This study sought to unravel the complex interplay between labor migration, induced poverty, and health outcomes from the perspective of Nepali migrant workers currently employed in Malaysia and GCC countries. The findings converge to present compelling evidence of a detrimental reality. They reveal that for many Nepali migrants, the pursuit of economic betterment through remittances is inextricably linked to a significant deterioration in physical and mental well-being, often perpetuated by systemic vulnerabilities and exploitative conditions that induce new forms of poverty. Our research, by centering the voices of migrants in situ, offers critical insights that extend, and at times challenge, existing understandings within the migration-development nexus and public health literature.

The “embodied costs” of labor resonate strongly with studies highlighting high occupational risks in sectors typically employing migrant workers [50, 51]. However, our findings move beyond statistical accounts of injury by illuminating the lived experience of differential safety standards and the normalization of risk. Participants’ perceptions of receiving substandard safety equipment due to their nationality—because they are “cheaper to replace”—suggest a deeply embedded devaluation of their labor [52, 53]. Framing these patterns through the lens of health capital [54] clarifies what is at stake: workers’ embodied stocks of physical and mental capacity are continually “depreciated” by hazardous tasks, long hours, heat exposure, and sleep disruption, and can only be “maintained” through adequate nutrition, rest, and timely care. The concept of “remittance‑induced health neglect,” where long‑term health is consciously sacrificed for immediate economic survival due to debt and family obligations, thus maps onto a coerced conversion of health capital into income flows, a dynamic often missed by studies focused on post‑return outcomes [39, 55, 56].

Within this lens, inadequate nutrition is not a peripheral hardship but a primary mechanism of accelerated health-capital loss. Participants described low wages and monotonous, starch-heavy employer-provided meals that left them calorie- and micronutrient-constrained relative to the metabolic demands of 10–12-hour shifts, especially in heat-exposed outdoor work. Under such conditions, nutrition—together with rest—functions as “maintenance investment”. When workers elect to defer the quality or quantity of food to enhance remittance flows, the reparative processes in their bodies are impeded; immune resilience is compromised, and the recovery from musculoskeletal strain is prolonged. This cycle of under-maintenance elucidates why seemingly “minor” health issues transition into chronic conditions and why the sentiment that “sickness is a luxury none of us can afford here” emerges as a rational adaptation within a context of severe economic coercion. Ultimately, the structural dynamics that depress wages, constrain dietary options, and penalize absenteeism institutionalize a relentless cycle of health capital depletion, thereby challenging prevailing narratives that dissociate remittance generation from the embodied costs involved in its production.

The findings from “The Invisible Chains” explore the significant mental health challenges faced by migrants, an issue that is frequently overlooked in economically focused discussions surrounding the migration‑development nexus. Here, we use *Invisible Chains* metaphorically to denote the cumulative, often unseen burdens—social isolation, remittance obligations, employer/visa dependency, and fear of job loss—that weigh on workers. Participants reported profound loneliness and anxiety related to remittance pressures, aligning with evidence on migration stressors and mental health outcomes [57]. These accounts critically examine the emotional labor undertaken by men in the context of transnational migration. This emotional labor encompasses the regulation and suppression of personal distress to “protect” family members, the performance of optimism during communication, and the management of financial requests. These practices are exacerbated by prevailing masculine norms that inhibit help-seeking behaviors. Within this demographic, emotional labor is not merely an ancillary aspect of the migration experience; rather, it is integral to the migration bargain itself. This dynamic redistributes psychological risk to the workers and exacerbates their isolation. Participants also described dehumanizing encounters when seeking healthcare. The articulated lack of accessible, culturally, and linguistically appropriate mental health services in destination countries points to a systemic failure at odds with the International Labor Organization’s (ILO) “decent work” agenda [58, 59]. The resulting “invisible” psychological costs affect productivity and safety and reverberate across households and communities—undermining family cohesion, children’s well‑being in transnational care arrangements, and community health upon return, sometimes with unaddressed trauma [60, 61].

The “Remittance Trap,” critically examines the paradoxical nature of migration for economic gain. Our findings strongly support the critique of the simplistic “triple-win” narrative of migration [62, 63]. The cycle of pre-migration debt, often facilitated by Nepal’s “fragmented migration sector” and exploitative intermediaries [33, 64], places workers in a state of debt-induced subservience from the outset. This finding, based on the current experiences of workers grappling with loans taken at exorbitant rates, offers a more dynamic understanding of “induced poverty” than studies focusing solely on remittance utilization for household improvement [65, 66]. The continuous siphoning of earnings through unfair deductions and wage theft, a reality exacerbated by power imbalances inherent in systems like the *kafala*—a sponsorship system prevalent in many Middle Eastern countries that legally binds migrant workers to a specific employer for the duration of their residency, severely restricting their mobility and ability to change jobs [67], further entrenches this economic precarity. The immense psychosocial burden of fulfilling remittance expectations regardless of personal hardship, leading to feelings of being a “money-making machine,” underscores a critical, often unquantified, health impact.

Finally, “Navigating Healthcare Deserts” illuminates the formidable systemic barriers to healthcare access. Participants reported prohibitive costs, perceived discrimination based on nationality and socio-economic status, and linguistic barriers. The insights from current migrants regarding their encounters with healthcare systems—where they often feel their concerns are dismissed or their value as patients diminished due to their national origin and occupational status—highlight an often-understated barrier that transcends mere financial constraints. This points to systemic biases and a critical lack of culturally and linguistically competent care, a dimension of healthcare inequity that our research brings to the fore, offering a more nuanced understanding. The “strategies of resilience” adopted by workers, such as self-medication or delaying treatment, do not indicate empowered agency but rather represent desperate coping mechanisms born from systemic neglect and the pressures of induced poverty. These often lead to the exacerbation of health problems, transforming treatable conditions into chronic illnesses, thus perpetuating a cycle of ill health and poverty upon return to their home country. This finding, capturing the decision-making process while in the destination country, offers a novel perspective on how barriers to timely healthcare access contribute to long-term negative health outcomes, directly linking migration experiences to the health-poverty nexus and underscoring the failure to uphold basic health rights as stipulated by international conventions [68–70].

### Limitations and future research

While this study offers critical insights into the health perspectives and induced poverty experienced by male Nepali migrant workers currently in Malaysia and GCC countries, we acknowledge certain limitations that open avenues for future research. A primary limitation is the exclusion of female migrant workers from our participant sample. Despite initial recruitment strategies designed for inclusivity and subsequent efforts through snowball sampling to reach female workers, we were unable to recruit any female participants. This absence is significant, as women often face distinct and compounded vulnerabilities within labor migration, including higher risks of gender-based violence, exploitation in feminized sectors like domestic work, and unique social stigmas [23, 25, 26]. Future research must, therefore, prioritize dedicated studies employing more nuanced and gender-sensitive recruitment approaches, such as collaborating with women-led Civil Society Organizations (CSOs), utilizing female researchers to build trust, and employing community-based participatory methods, to specifically explore the health experiences, economic precarity, and coping mechanisms of Nepali women migrant workers in situ. Understanding their perspectives is crucial for developing gender-sensitive policies and interventions, particularly given the increasing feminization of certain migration corridors [28, 71]. Furthermore, these insights are primarily from male workers in specific sectors within Malaysia and several GCC states; experiences may differ significantly in other destination countries or occupations. Future research could employ mixed-methods approaches and larger, more representative samples to quantify the prevalence of the health issues and economic challenges identified here, and to explore variations across different demographic groups and migration corridors.

### Recommendations and conclusion

Based on the realities voiced by Nepali migrant workers in this study, several actionable recommendations emerge, aimed at mitigating induced poverty and safeguarding migrant health. First, the Government of Nepal must urgently strengthen its pre-departure regulatory framework by mandating the “employer pays” principle for all recruitment costs to dismantle the cycle of debt-induced subservience, and ensuring that pre-departure orientations are comprehensive, and address specific occupational and health risks, including mental well-being. Second, robust bilateral agreements with Malaysia and all GCC states are imperative; these must include provisions for regular workplace inspections, accessible and culturally competent healthcare services for migrant workers, effective grievance redressal mechanisms that are independent of employers, and establish clear, joint monitoring bodies with representatives from both sending and receiving states. Third, destination countries must be urged to reform restrictive systems like kafala that entrench worker dependency and facilitate exploitation, and to ensure their labor laws and safety standards are stringently applied to all migrant workers, irrespective of nationality or sector. In addition, destination-country policy should institute basic social protection floors so that workers are not compelled to work when ill, including statutory paid sick leave and sick-pay entitlements; non‑retaliatory, job‑protected medical leave (with protection from dismissal or deportation due to illness or injury); mandatory workers’ compensation and wage‑protection schemes; and enforceable rest‑break and heat‑stress safeguards. Finally, international organizations and civil society organizations (CSOs) should collaborate to enhance independent monitoring of labor conditions, provide direct support services to migrants in distress, and advocate for systemic reforms at both national and international levels; additionally, destination countries should create clear pathways for legal recourse that are genuinely accessible to migrant workers, including for those who may become undocumented as a direct result of employer abuse or contract violations, thereby addressing power imbalances and fear of deportation.

This study, through the firsthand accounts of male Nepali migrants, reveals the profound and multifaceted health costs embedded within the contemporary labor migration system. The concept of “induced poverty”—manifesting as debt, precarious living and working conditions, and constrained access to healthcare—emerges as a central determinant of health, inextricably linked to the pressures of the remittance economy. Our findings challenge overly optimistic portrayals of migration as a purely developmental tool, instead highlighting how systemic failures in governance, both in Nepal and in destination countries, contribute to the erosion of migrant workers’ physical and mental well-being. By foregrounding contemporaneous, in‑situ accounts, this study underscores the urgency of reorienting migration governance toward frameworks that center human rights, health, and dignity.

## Supplementary Information

Supplementary Material 1

## Data Availability

The data supporting this study’s findings are available on request from the corresponding author. However, the data is not publicly available due to privacy or ethical restrictions.
