# Supplementary material for: Induced poverty, increased remittances: unveiling the lived realities of Nepali migrant workers
Source: BMC Public Health. 2025 Nov 27;25:4178. doi: 10.1186/s12889-025-25503-0 (PMC12659327; doi:10.1186/s12889-025-25503-0)
Supplement: Supplementary file 1 — Supplementary Material 1 [file 12889_2025_25503_MOESM1_ESM.docx]

**Table 1: Semi-structured Interview Guide Outline**

| Question No. | Interview Questions |
| --- | --- |
| 1 | Can you tell me about your journey to [Country of Destination] and what motivated you to seek employment here? |
| 2 | Could you describe your typical workday, including the nature of your work, the hours, and the physical demands involved? |
| 3 | How would you describe your current living conditions, including your accommodation and access to food and sanitation? |
| 4 | What are the biggest challenges or stressors you face related to your work and living situation here? |
| 5 | How has your physical health been since you started working here? Have you experienced any work-related injuries or illnesses? |
| 6 | Could you talk about your experiences with accessing healthcare services in [Country of Destination] if and when you have needed them? |
| 7 | How does the pressure to send remittances back to your family in Nepal affect your daily life, financial decisions, and overall well-being here? |
| 8 | Have you taken loans to travel abroad? If so, could you share your experience with this and how it impacts your current financial situation? |
| 9 | Considering the separation from family and other peers, how would you describe your mental and emotional well-being while working here? |
| 10 | In what ways do you feel your health or well-being has changed – for better or worse – as a result of your migration experience? |
| 11 | If there was one thing you could change about the migration system or your working conditions to improve your health, what would it be? |
